# What constitutes successful commissioning of transition from children’s to adults’ services for young people with long-term conditions and what are the challenges? An interview study

**DOI:** 10.1136/bmjpo-2017-000085

**Published:** 2017-09-11

**Authors:** Niina Kolehmainen, Sara McCafferty, Gregory Maniatopoulos, Luke Vale, Ann S Le-Couteur, Allan Colver

**Affiliations:** Institute of Health and Society, Institute of Health and Society, Newcastle University, Newcastle upon Tyne, UK

**Keywords:** health services research, health economics, general paediatrics, qualitative research

## Abstract

**Objective:**

We explored what constitutes successful commissioning for transition and what challenges are associated with this. We aimed: (1) to identify explicit and implicit organisational structures, processes and relationships that drive commissioning around transition; (2) to identify challenges faced by commissioners; and (3) to develop a conceptual model.

**Design:**

A qualitative interview study.

**Setting:**

Commissioning and provider organisations across primary and secondary care and third sector in England, UK.

**Participants:**

Representatives (n=14) from clinical commissioning groups, health and well-being boards and local authorities that commission national health services (NHS) for transition from children’s to adults’ services in England; NHS directors, general practitioners and senior clinicians (n=9); and frontline NHS and third sector providers (n=6).

**Results:**

Both commissioners and providers thought successful transition is personalised, coordinated and collaborative with a focus on broad life outcomes and actualised through building pathways and universal services. A multitude of challenges were described, including inconsistent national guidance, fragmented resources, incompatible local processes, lack of clear outcomes and professional roles and relationships. No single specific process of commissioning for transition emerged—instead complex, multi-layered, interactive processes were described.

**Conclusions:**

The findings indicate a need to consider more explicitly the impact of national policies and funding streams on commissioning for transition. Commissioners need to require care pathways that enable integrated provision for this population and seek ways to ensure that generalist community providers engage with children with long-term conditions from early on. Future research is needed to identify a core set of specific, meaningful transition outcomes that can be commissioned, measured and monitored.

**What is already known on this topic?**

- Young people with long-term conditions who transition from children’s to adults’ services have negative experiences of healthcare and poor health and social outcomes.
- Despite policy and guidance, the transition process remains fragmented and is a key risk period for poor clinical outcome.
- Quality of transition can be affected by commissioning, that is, how services are planned, contracted and monitored, but there is a paucity of evidence about commissioning for transition.

**What this study hopes to add?**

- Commissioners and providers thought successful transition is personalised, coordinated and collaborative with focus on broad life outcomes and actualised through building pathways and universal services.
- Challenges to commissioning for successful transition include inconsistent national guidance, fragmented resources, incompatible local processes, lack of clear outcomes and professional roles and relationships.
- Recommendations include focus on coordinated pathways for this population, engaging generalist community providers from early on and identifying core transition outcomes for commissioning and monitoring.

## Introduction

In the UK, more than 25 000 young people with long-term conditions transition from children’s to adults’ services every year.^1^ Many of them have negative experiences of healthcare during transition and poor health and social outcomes following transition. Furthermore, despite 20 years of policy and guidance, the improvements in transition are limited. The process remains fragmented and is a key risk period for poor clinical outcomes.^2–6^

Long-term conditions in young people refer to conditions that cannot be cured with current interventions but that can be managed. These include, for example, diabetes, asthma and developmental disabilities. The transition of young people with long-term conditions from children’s to adults’ services can be affected by a range of factors. One of them is commissioning,^7 8^ the process by which public services are planned, contracted and monitored. It is widely, internationally recognised that, to understand and improve any service provision, including transition process, the functions of planning, contracting and monitoring need to be understood.^9^

There is currently a paucity of peer-reviewed evidence about commissioning for transition; our systematic review found no published papers (online supplementary file S1). The present study is the first to contribute evidence on this topic and through that to inform practice and guidance on commissioning for transition. The study explored what constitutes successful commissioning for transition and the challenges associated with this. The objectives were: (1) to identify the explicit and implicit organisational structures, processes and relationships which drive commissioning around transition; (2) to identify the challenges faced by commissioners; and (3) to develop a conceptual model.

10.1136/bmjpo-2017-000085.supp1 Supplementary file 1 f1

While there are differences in health systems in terms of commissioning, it is also likely that there are also shared points of learning. UK health system provides one diverse set up from which such learning can be obtained. In the UK, over the last 20 years, there has been a fundamental separation of the bodies that commission services from the bodies that provide them. Some specialised services are commissioned centrally, and more general services are commissioned by local groups with strong representation from primary care and local authority. The process of commissioning involves assessing needs, deciding priorities and strategies and then buying services on behalf of the population from providers such as hospitals, clinics and community health bodies. It is an ongoing process, where the commissioners constantly respond and adapt to changing needs and circumstances.

## Methods

This was an interview study, using conversational techniques to gather data, within a 5-year Transition Research Programme funded by the National Institute for Health Research (RP-PG-0610–10112) to generate evidence for commissioning and provision of better transition for young people with long-term conditions. This interview study received ethics approval from the Newcastle University Faculty of Medical Sciences Ethics Committee (ref: 00767/2014).

### Setting, sample and recruitment

Interviewees were sampled using purposive and snowball sampling from two areas in the North of England and from national leaders across England, including from: clinical commissioning groups, health and well-being boards and local authorities that commission national health services (NHS) transition from children’s to adults’ services in England; NHS directors, general practitioners (GPs) and senior clinicians with roles relevant to transition; and frontline NHS and third sector providers. First, the study steering group nominated potential interviewees; the nominees were then considered for participation based on their job title; those selected were emailed a letter inviting them to participate; and if no response was received, then up to three follow-up attempts were made by telephone. Interviewees who were approached were also invited to nominate further interviewees, and recruitment continued until new data no longer added content. We anticipated that around 25 interviews would result in sufficient coverage of a range of views across contexts. Informed written consents were taken.

### Data collection

The interview schedule was based on modified critical incidence technique,^10^ informed by grey literature (online supplementary file S1) and conversations with the research team and the steering group. The interview schedule (table 1) was designed to encourage participants to reflect on successful and unsuccessful practices for commissioning in the context of transition and to cover perceptions of (i) the organisational structures, processes, relationships, barriers and facilitators related to commissioning and (ii) the relative influence of policy drivers, relationships with providers and external influences. SMC, a researcher with PhD in healthcare commissioning, conducted the interviews either face to face in interviewee’s chosen setting or by telephone. Interviewees had no prior knowledge of or relationship with the interviewer. The interviews lasted a median of 45 min, with a range of 27–68 min, and were conducted from April 2014 to August 2014, audio-recorded verbatim and later transcribed. Three interviews were conducted by phone, the remainder face to face.

**Table 1:** Interview schedule

Background/context	
1	Can you tell me what you understand by the term ‘transfer’ or ‘transition’ in healthcare? How would you define a ‘successful transition’?
2	Can you tell me about your role and: (a) How you are or have been involved in transition? (b) How you are or have been involved in commissioning?
Successful commissioning outcomes	
3	Can you describe an example of when transition or commissioning for it has been undertaken successfully? (Outcomes)
Successful commissioning activities/processes	
4	With respect to the example shared can you describe the activities, actions or processes that were undertaken to achieve this outcome?
Unsuccessful commissioning outcomes	
5	Can you describe an example of when transition or commissioning for it has been undertaken unsuccessfully? (Outcomes)
Unsuccessful commissioning activities/processes	
6	With respect to the example shared can you describe the activities, actions or processes that were undertaken which resulted in this outcome?
Any other points	
7	Are there any other issues which you consider to be relevant that you would like to discuss?

The interview schedule was piloted with ALC who had both clinical and academic experience of transition and commissioning. The interview guide was designed to use open questions, which were used dynamically (as described in table 2). Questions were not adapted for different roles; rather the use of probes was tailored to fully elicit different experiences between roles.

**Table 2:** Quality assurance techniques employed

Credibility	During the data collection, contact was established through demonstrated interest in the responses, attentive listening, understanding and respect for what the participant says^19^
	The sequencing and posing of questions was carefully considered and was dynamic so that the questions promoted positive interaction between the participant and the interviewer and stimulated the participant to share their experiences and points of view^19^
	All interviews included an opportunity for participants to comment on any topic covered in the interview or any new topic that they felt was relevant^19–22^
	Triangulation: accounts between participants were compared and contrasted
	Member checking: the themes and their content were shared and discussed with the study steering group
	Frequent debriefing: study progress, methods, emerging themes and any issues were reported to and scrutinised by the research programme senior team at regular intervals
Transferability	The sampling frame and criteria (see the Methods section) and the key population characteristics (see the Results section) were clearly recorded and reported
Dependability and confirmability	To allow a nuanced, multifaceted analysis and reconciliation of any tensions in the coding and concepts, researchers from different disciplinary backgrounds with different expertise contributed to the data analysis, including: GM, sociology; NK, behaviour change, NHS practice in long-term conditions; SMC, commissioning, health economics; AC, paediatrics; ALC, child and adolescent psychiatry; LV, health economics; and DR (in acknowledgements), NHS management
	Involvement of several researchers with different viewpoints and expertise also helped to ensure that the framework was adapted to reflect the data rather than making the data ‘fit’ the framework
	Involvement of new researchers (GM, NK) in the data analysis encouraged further peer examination through critical discussion
	Audit trail: researchers kept field notes (SMC) and a logbook of data analysis (GM, NK) and established an electronic data analysis and synthesis trail of the development of the themes

NHS, national health services.

### Data analysis

The transcripts were analysed using framework analysis.^11^ Framework analysis allows both emergent data themes and the explicit recognition and use of a priori issues in the analytical framework. Framework analysis is increasingly being used within health services research, and it fitted the aims of our study as we had predefined areas we wished to investigate while remaining open to the emergence of further topics and themes. A series of interconnected steps within the framework approach describes the processes that guide the systematic analysis; these steps allow an iterative refinement of themes and are described below.^12^

An initial conceptual framework based on literature and researchers’ experiential knowledge was expanded and modified in iterative cycles using themes emerging from the data. This produced a cumulative, refined framework that integrated the initial conceptual framework and the study results. The specific steps were as follows. *Familiarisation:* two researchers (GM, NK) developed an initial sense of the data by reading through a sample of transcripts. *Identifying the initial coding framework:* three researchers (GM, NK, SMC) independently recorded their impressions and deductive themes. GM and NK discussed these impressions, related them to their previous knowledge and expertise and agreed on the initial conceptual framework. This process was repeated for six rounds, with the two researchers reading further transcripts between each discussion round. The discussions consisted of the researchers talking through the emerging issues, themes and relationships and agreeing on themes, codes and relationships, which were added to the framework. *Indexing:* once the framework became stable (ie, few modifications were required on each round), GM used it to ‘index’ the remaining transcripts one by one. This involved ‘sifting and sorting’ the remaining data and allocating these into the coding framework. The researcher took notes of any changes to the framework and issues, and these were discussed with NK. This process was repeated until all data were indexed and the final framework agreed upon. *Charting:* data from the transcripts were summarised according to the themes and codes (‘categories’) to reduce the data while carefully retaining the original meanings. References to illustrative quotations were tagged and managed using Microsoft Word and NVivo10.

### Quality assurance

We employed recognised quality assurance techniques^13^ to ensure credibility, transferability, dependability and confirmability (table 2).

## Results

Forty-six interviewees were approached; 29 agreed to participate. The participants covered a range of roles across the target population (table 3). Reasons for non-participation were: no response to email or email follow-up, change in role and lack of time to participate.

**Table 3:** Summary description of the participants

Coverage	Participant role	Organisation(s)
Regional	Commissioners at different levels of seniority and related managers (n=10)	Health and social care commissioning organisations, including local authorities, commissioning support units and clinical commissioning groups
	NHS director/manager (n=2) NHS clinicians (n=4) General practitioners (n=3)	NHS
	Transition planning workforce (n=2) Transition managers, coordinators (n=2)	Local authority
National	Clinical leaders (n=3)	NHS and NHS England
	Voluntary sector leaders (n=3)	Charities providing care

NHS, national health services.

### Successful transition

While no single definition of successful transition emerged, some key characteristics were described (table 4). These included that transition should: (i) be personalised; (ii) be planned, coordinated and collaborative; (iii) focus on broad developmental and life outcomes; (iv) build pathways from children’s services to adults’ services rather than just rely on individual single solutions; (v) ensure coordination and continuity of relationships and knowledge across sectors and life domains rather than just transfer young people from one service to another; and (vi) use universal services such as GPs where possible with tailored enhanced support as required.

Box 1 **Selected quotes about perceived characteristics of successful transition**

**Personalised, planned, coordinated, collaborative with focused on broad developmental and life outcomes:**

“(…) what all the legislation is telling us, and all the national direction is about, is about personalisation. (…) [in current practice] we keep on just focusing on the here and now. What we should be doing is (…) predict what the needs will be in the future (…)” (Commissioner/related manager 1, Regional)

“(….) a smooth journey and needs met. (…) the much wider picture. So your health needs will impact on your employment outcomes or your education (…) and what you do with your aspirations within your community (…)” (Member 1 of transition planning workforce, Regional)

“(…) I think successful transition (…) has to be addressed and introduced as a concept at the age of 14+ school review (…) then the families, and the young people, and the professionals begin, hopefully, to develop some type of joint work between them (…)” (Voluntary sector leader, National)

**Builds pathways, ensures continuity and uses universal services:**

“(…) if the systems were right, so if you had children’s services interfaced properly into adult services there was a clear pathway (…) children would just sort of flow through (…)” (Commissioner/related manager 1, Regional)

“(…) a successful transition is where the person undergoing transition has the change of care seamlessly, without any interruption in their therapeutic relationship, in their treatment strategy, and in their engagement. (…) the aspects of continuity, information continuity, relational continuity, therapeutic continuity.” (National health services clinical leader 1, National)

“(…) this concept of universal (…) you might have somebody who’s complex and needs [Children and Adolescent Mental Health Services] and learning disability team or whatever, and has some physical needs as well. But still can access the already commissioned services. And if they interfaced well then the transition could be seamless and wouldn’t need active commissioning. (…)” (Commissioner/related manager 1, Regional)

### Challenges

Four meta-themes related to challenges emerged: (1) the broad context: legislation, policy and wider life transitions; (2) structures, processes, pathways and relationships; (3) service-level coordination, sign-posting and relational support; and (4) outcomes and contract evaluation.

#### The broad context: legislation, policy and wider life transitions

Participants discussed a range of features related to national legislation and national and local policies that they perceived to influence commissioning and healthcare. One common theme was service eligibility. Participants described problems in relation to the criteria commonly used for service eligibility, including age, severity and diagnosis. Inconsistencies within and between sectors in cut-offs created challenges for effective commissioning.

(…) some services will say, “We go up to 16”, some go up to 18, some go up to your 19th birthday, some go up to 25, and some are lifelong (…) depending on who you are and what service you’re dealing with depends on what, even age group, you’re dealing with in terms of transition. (Commissioner/related manager 2, Regional)

(…) mental health conditions that children suffer from do not actually make the grade for adult mental health services. (…) (Clinical leader, National)

Participants also consistently highlighted that young people’s lives and transitions are wider than the prescribed service remits and described challenges stemming from a reductionist approach, which requires partitioning the wider life to public sector remits.

(…) [the Government] send guidance on what they think a health need is and what an education need is, or a social care need is, which again creates barriers. So, for instance, if you are peg-fed when you’re at home you could say that’s a healthcare need because you need to be fed to live. While you’re at school, school are responsible for making sure you can access education; you can’t access education if you’re hungry; so is it then [education’s] responsibility to feed you? (…) (Commissioner/related manager 4, Regional)

Legislation that requires more coordinated public services was hoped to address the segmentation, but participants also expected the impact of any legislation to be hampered by fragmented resource allocation.

(…) Government, is saying, “Well we need to go through to 25” that’s fine, but as long as there’s an 18-year-old cut-off and there isn’t the funding… the world isn’t going to change. (Commissioner/related manager 4, Regional)

Some participants suggested that joint commissioner posts, funded together by health and local authority, could facilitate positive arrangements.

(…) my role (…) it’s half funded by the local authority (…) I think the principle of a joint post is good, (…) children in education, there’s links to social care; it’s all a very interlinked (…) [I resolve funding disputes] particularly between the local authority and the [health] about health need or a social need, and who should pay (…) (Commissioner/related manager 4, Regional)

However, others expressed a belief that transition is not a government or commissioning priority, and there is limited willingness to allocate resources for transition.

(…) while we’re aware that [transition] is an issue, we’re also acutely aware that there are bigger issues at stake (…) you tend to find that the big issues, like the fact that we’re about, potentially, about £8 million short in terms of budget this year is much more of a priority than transition (…) (Commissioner/related manager 5, Regional)

#### Commissioning structures, processes, pathways and relationships

Overall, participants described that the multitude of local structures, processes and agencies involved in commissioning and provision created a major challenge.

(…) with CCGs [Clinical Commissioning Groups] and commissioning support, with NHS England having their role, with public health being in the council, with the different bits of the council, the education bit and the care bit. (…) Responsibility, process, who to talk to, who, who is doing what. (…) (Commissioner/related manager 4, Regional)

Other challenges repeatedly described were that the services for children and adults are commissioned separately and on different organising principles, including differences in clinician roles.

(…) [in transition] the paediatrician is referring to an adult respiratory doctor, an adult gastroenterologist and an adult neurologist to replace [the paediatrician] (…). [who] may have dipped in and out of the paediatric specialities in those areas (…) (NHS clinician 2, Regional)

These differences resulted in ‘the gap’, a situation where there was no clear destination for the young person to transfer to. Commonly described approaches to fill the gaps were the use of personalised, tailored solutions for individuals and personalised budgets. In contrast, examples of proactive commissioning of pathways for populations were rare, and some explicitly recognised this.

(…) the way that commissioning works currently is that (…) usually the providers identify gaps and they’re then asked to fill those gaps within, usually within the resource or something you get a little bit of extra resource to do that. Erm, but that’s not the same as commissioning a full pathway from start to finish (…) (NHS Director 1, Regional)

Commissioning successfully without gaps was perceived to be greatly facilitated by effective relationships and communication that fostered trust and good faith across stakeholders.

(…) we had a very good commissioning team at the time (…) [the commissioners] worked in the same building. So they had a very good understanding of transition and the gaps (…) the children’s commissioner worked alongside the adult commissioner (…) once we’d established that good relationship with the adult commissioners we’ve built on that year in year out. (Voluntary sector leader, National)

#### Service-level coordination, sign-posting and relational support

Coordination, sign-posting and relational support were consistently discussed as central to successful transition. Proposals for commissioning solutions to achieve these focused on enabling young people to self-manage their condition and care with support of a nominated professional. One common proposal was to involve GPs more proactively from early on, alongside paediatricians.

(…) parents build up great relationships with these paediatricians and so, if they’ve got, any queries, regardless of whether it’s associated to that child’s disability or not, obviously they’re going to ring the person who knows them best and is, kind of, in charge of their care. So for me that’s a really big risk for transition because you’ve had this brilliant service from this one particular person, for the whole of your child’s life, and when they’re approaching transition there’s no equivalent (…) (Commissioner/related manager 9, Regional)

(…) I think we need to involve the GPs from very much earlier on. (…) maybe if you involved the GP, gives the confidence to the families as well. (…) (NHS clinician 3, Regional)

Other proposals, for improving transition, included use of specialist nurses and other community clinicians and the creation of ‘transition workers’.

(…) identifying the children and young people at around [age] 14, 15 then the transition workers will introduce themselves and begin to get that process in place (…) (General practitioner 1, Regional)

#### Outcomes and contract evaluation

Participants emphasised that transitions should be outcomes focused and these outcomes should be considered broadly across life domains. However, participants’ accounts lacked specific examples of outcomes-based commissioning. Instead, they conveyed difficulties in specifying outcomes, and some participants explicitly said it was difficult to identify clear, shared values and outcomes for commissioning for transition.

(…) outcomes based commissioning (…) with health, I mean outcomes are so often, you know, they’re not, (…) it depends on how you determine or define the outcomes (…) it is very difficult to (…) I think everyone, yeah, sort of talks around outcomes based commissioning as a good idea and it is better than kind of just throughput. Erm, erm but, er, (…) it still feels a bit too hard to do and there isn’t this kind of universal understanding of what that is and what it means, let alone how you measure it. (Commissioner/related manager 11, Regional)

(…) it is so complicated and it’s so multiagency (…) we don’t have a shared value base of what we’re trying to achieve with young people and their families. (…) commissioning circles, lovely things they are, but they don’t mean anything to young people and their families. (Laughter) (…) (Member of transition planning workforce 3, Regional)

Similarly, contract management based on outcomes, as opposed to activity, was perceived to be difficult.

(…) you look at it within the contractual management (…) [Historically] commissioning has been very much [about] (…) number of contacts, number of review appointments, maybe even staffing numbers (…) all the kind of rhetoric and theory around commissioning for outcomes (…) everybody talks about it all the time, but to actually make it meaningful (…) it’s relatively easy to measure activity; it’s very hard to measure outcomes. (…) (Commissioner/related manager 4, North England)

One way participants sought to evaluate outcomes was through generic feedback from service users, but this too was perceived to have limitations, for example, providers failing to collect this data.

### Conceptual model of commissioning for transition

**Figure 1:**
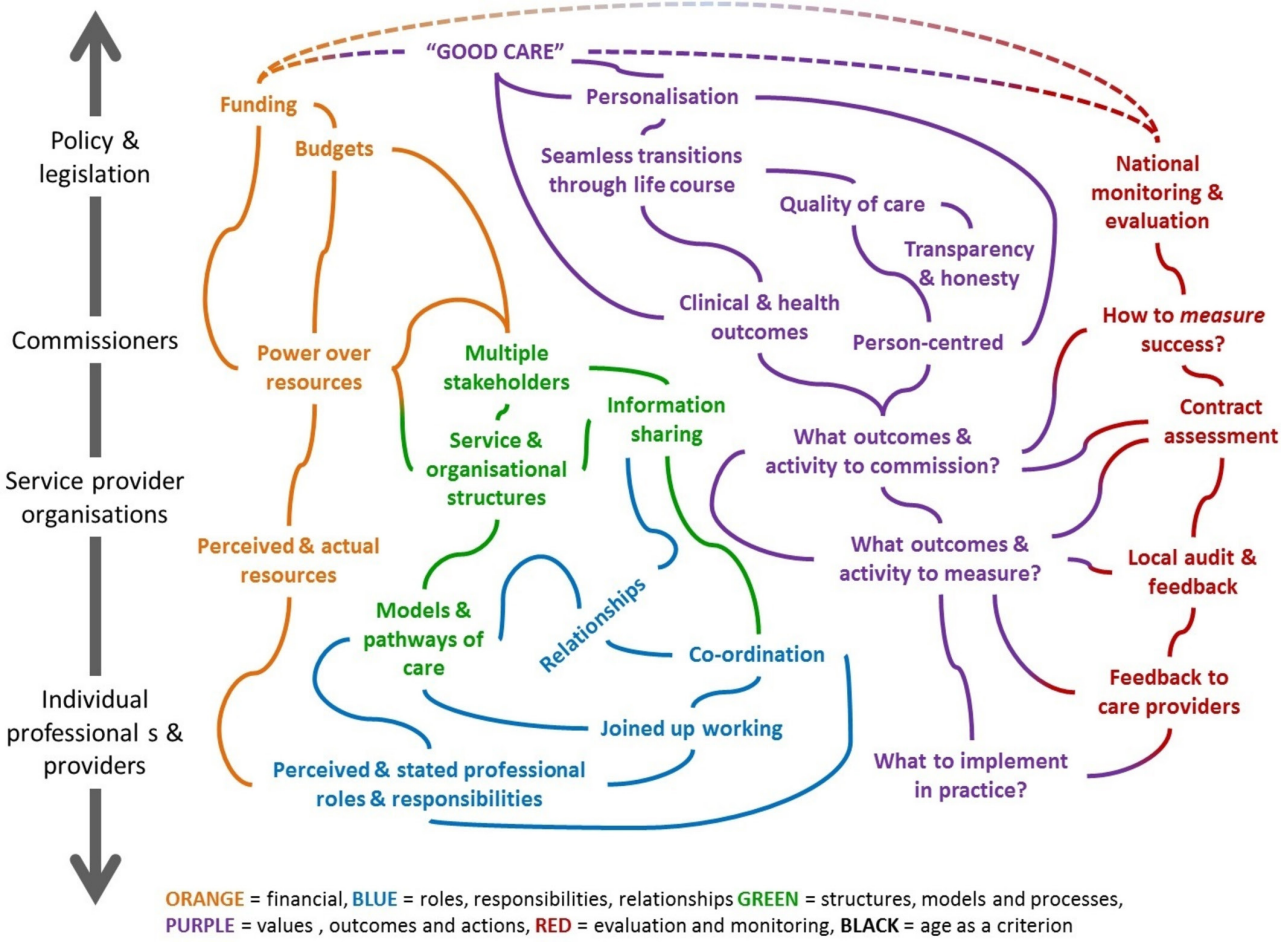
A visual summary conceptualising the process of commissioning for transition as it emerged from the data analysis.

While the participants provided rich reflections on key characteristics and challenges, there was limited discussion about any unique steps related to commissioning for transition (as opposed to commissioning in general). There was little evidence of a specific ‘transition process for commissioning transition’. Instead, the accounts reflected complex and nuanced processes entangled with other local and organisational structures, processes and relationships as described above. Figure 1 provides a summary output of the results in terms of stakeholders’ perceptions of the organisational structures, processes and relationships that drive commissioning for transition. It illustrates the interrelated nature of the themes that emerged and reflects the complexity of the commissioning process as described by the participants.

## Discussion

This study found that both commissioners and providers believe transition from children’s to adults’ services should be personalised, coordinated and collaborative with focus on broad life outcomes and that such transitions should be realised through building pathways and universal services where possible. However, a multitude of challenges were described in relation to commissioning for such transitions including inconsistent national guidance, fragmented resources, incompatible local processes, lack of clear outcomes and professional roles and relationships. No single, specific process of commissioning for transition emerged—instead complex, multi-layered, interactive processes were described.

Commissioners identified clearly the inevitable tension between the need to commission for personalisation of healthcare and at the same time securing pathways of care. There is no easy solution to this. One option is for the responsibility for personalisation to lie mainly with the service providers while commissioners set the required pathways of care by purchasing the necessary staff and facilities.

The study used established qualitative methods, with clear quality assurance strategies, which provide confidence in the findings. Interviews continued until data saturation was reached, between subgroups of participants and in general. The data on commissioning processes specific to transition were thin. It is possible that this is a true finding, that is, that there are no steps unique to commissioning for transition or that a different elicitation method would have yielded richer data with a different finding. We did not approach young people to ask their views about commissioning because, in the UK, commissioning is very separate from service provision and patients for the most part only experience services. Young people in transition have the major task of gradually taking responsibility for their own healthcare in the context of the services available; we thought it unlikely that they would have knowledge of commissioning. Efforts have been made to engage the public in the work of commissioners, but this has been very difficult and especially difficult to engage adolescents in discussions about commissioning.^14^

Our findings concur with other stakeholders’ views on the criteria for successful transition and further elaborate these by identifying some of the barriers to commissioning for such transitions.^15^ For example, the findings on fragmentation in funding and the tendency to commission individual, single solutions as opposed to pathways provide possible explanations for the ‘gap’ between children’s and adults’ services reported in studies with service users. Notably, these types of learnings are likely relevant across commissioning systems and thus have the potential to inform commissioning beyond the study setting of the UK.

The findings have a number of implications. These include a need for policy makers to facilitate joint funding arrangements across sectors and to be aware that using chronological age as a criterion risks creating barriers to effective commissioning. Commissioners need to reflect on the tendency to fund single solutions rather than create care pathways and to consider incorporating available legislation (such as the Children and Families Act 2014 in the UK) in service specifications and contract monitoring to encourage more integrated services. Commissioners may also wish to seek ways to ensure that GPs or other community providers are involved with children with long-term conditions from early on in order to be better placed for coordinated adult care.

The extent of difficulties in identifying specific outcomes that should be commissioned, measured and monitored indicates a need for research to develop a core set of agreed transition outcomes with related measures. Previous research, for example, on benchmarks for transition^15^ and on commissioning for long-term conditions,^14^ as well as guidelines for good transition practice,^16^ provides a starting point. There have also been two useful Delphi exercises.^17 18^
