# Supplementary material for: What constitutes successful commissioning of transition from children’s to adults’ services for young people with long-term conditions and what are the challenges? An interview study
Source: BMJ Paediatr Open. 2017 Sep 11;1(1):e000085. doi: 10.1136/bmjpo-2017-000085 (PMC5673067; doi:10.1136/bmjpo-2017-000085)
Supplement: Supplementary file 1 [file bmjpo-2017-000085supp001.pdf]

Supplemental file S1: A review of literature about commissioning transition

A systematic review of peer-reviewed literature relevant to commissioning for transition was conducted to inform this study. The core bibliographic databases used were: Medline (1996-), Web of Knowledge (WOK) (1996-) and Scopus (1996-). A structured search strategy was formulated using controlled search terms, including free text terms and MESH headings where available. Given the complexity and ambiguity of the terms 'commissioning' and 'transition', all alternative terms were used in order to be as thorough as possible. Search terms (Box S1) were applied across databases. To ensure that the literature review remained up to date, searches were conducted regularly over the duration of the review from September 2013 to March 2017. The identified papers were reviewed using explicit exclusion criteria (Box S2).

The PRISMA diagram (Figure S1) shows how the searches of Medline and WOK databases were structured. Separate searches for papers on commissioning and transition identified 302 papers relevant to both topics, which were then screened and assessed for eligibility according to the exclusion criteria. The most common reason for exclusion was that the paper focused only on transition, with no content about commissioning. No academic publications on commissioning for transition were identified.

Box S1. Academic literature search

<u>Commissioning search terms</u>
"Delivery of Health Care" OR [(Contract Services OR Contract* OR Commission* OR fund* OR purchase* OR budget* OR plan* OR provide* OR manage* OR procurement* OR assess*NEAR/1needs) AND healthcare]

<u>Transition search terms</u>
Transition to Adult Care OR Health Transition OR transition* OR continuity* OR pathway OR "service mapping" OR transfer*

AND healthcare AND ('to adult OR 'from paediatric') AND (young person* OR young adult* OR adolescent* OR youth OR child*)

Box S2. Academic literature exclusion criteria

<u>Title and abstract criteria</u>

1. Non-English Language
2. Transitions other than from children’s services to adult services
3. Populations other than young people with ‘complex health needs’ as defined for the research programme
4. Only identify current problems to transition, no mention of approaches to improve transition or barriers to successful transition

<u>Full text criteria</u>

1. It was a book
2. Not an academic or peer reviewed research study
3. Transition papers with no focus on commissioning (or related) processes
4. Focus of the paper not on commissioning or transition processes in the NHS

Figure S1. Academic literature PRISMA diagram

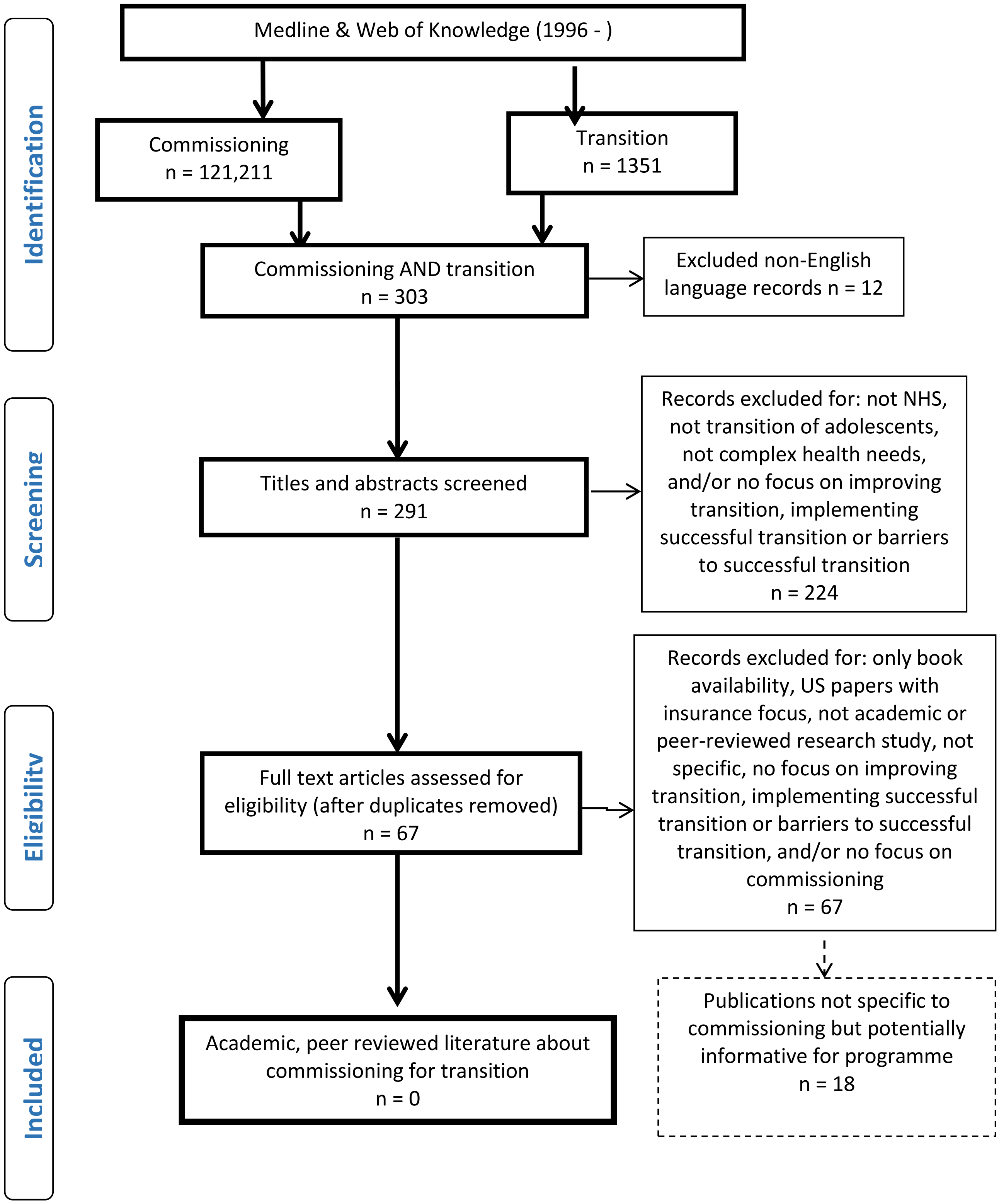

The lack of peer-reviewed papers about commissioning for transition led us to undertake a further grey literature search. By grey literature we understood “information which is produced on all levels of government, academics, business and industry..., but which is not controlled by commercial publishers".[1] It is more broadly described as material not published commercially or indexed by major databases,[2] e.g. policy papers, discussion papers, briefings not covered in the bibliographic search. The same search terms used for the peer-reviewed literature were applied wherever possible (Box S3). We hand-searched the following sources: Social Care Institute for Excellence (SCIE), Health Management Information Consortium (HMIC) (Department of Health, Kings Fund), Nuffield Trust, Public Health England (PHE), NHS Evidence (NICE), NHS Institute for Innovation and Improvement, NHS Improving Quality, Health Services Management Centre (HSMC), National Research Register Archive, UK Clinical Research Network, and Google Scholar. These searches were further supplemented using backward and forward searching techniques [3] on any key literature, looking at its references, citations and other papers written by same authors. ‘Snowball’ referencing was used to retrieve references cited in key primary research texts. Papers were also retrieved from email subscription, discussion listings, and supplemented by literature obtained within the wider programme of research. The identified literature was screened for inclusion using exclusion criteria (Box S4). The search of grey literature was completed in June 2014; we decided to keep the search open and update it throughout the study up to July 2016.

Box S3. Grey literature search strategy

SCIE search terms

'Commissioning for Transition' – simple search box didn't permit combining terms with 'AND' / 'OR' in same manner as Academic database search.

HMIC search terms

'Commissioning' control vocabulary selected:

Locality Commissioning OR General Practice Commissioning Groups OR Practice Based Commissioning OR Commissioning Agencies OR Hospital Commissioning OR Lead Commissioning OR Commissioning

AND

'Transition' control vocabulary selected:

Palliative care OR "Quality of patient care" OR Patient transfer OR transition to adult services OR Mental health services OR Learning disabilities

AND

Management practice OR Primary Care Trusts OR Healthcare OR Primary care OR Organisational culture

AND

'Adults' or 'Young People'

Nuffield Trust and PHE search terms

Hand searched publications on website under Commissioning topic where possible

Box S4. Grey literature exclusion criteria

1. Non-English Language
2. Non-comparative health care system – developing countries
3. Older populations
4. Lower grade cases, or non-disability cases e.g. dentistry
5. Lack of commissioning or lack of transition content
6. Only book availability
7. Focus of the paper not on commissioning or transition processes in the NHS
8. Lack of focus on health care (purely social or educational services)

Figure S2. Grey literature PRISMA Diagram

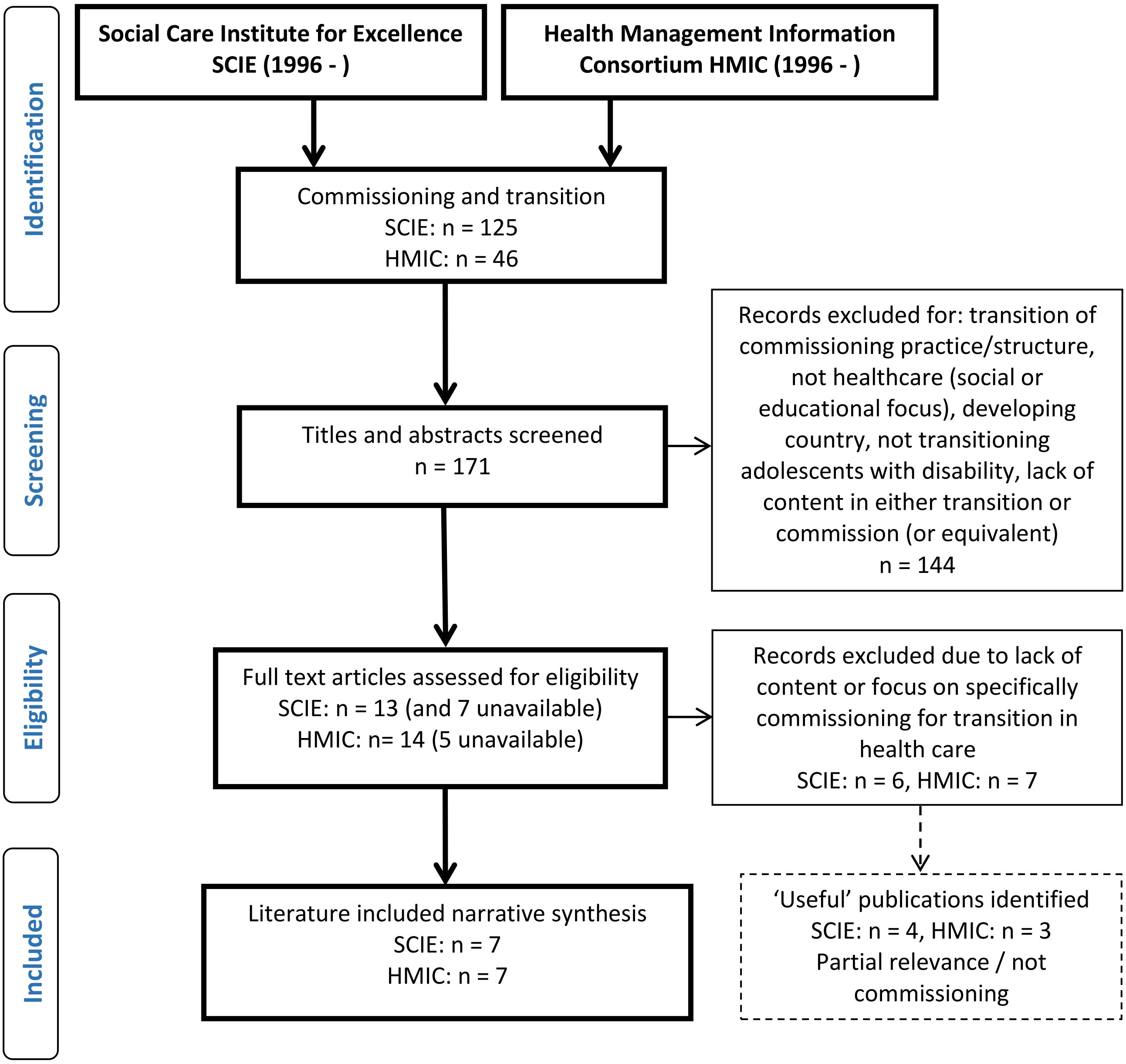

The review of grey literature identified two main areas literature: commissioning, and organisation and delivery of care.

1 Commissioning

Within the grey literature, no specific policies relating to commissioning for transition were identified. However we identified some key themes from policies with a focus on improving outcomes for children and young people, key delivery plans with a focus on personalisation of care, and some commissioning guidelines. Together these themes may provide useful insight into some of the issues which may be relevant for commissioning for transition. The

four sub-themes related to commissioning were: transition protocols, clinic structure, funding, and co-ordination.

1.1 Transition protocols

A number of recommendations were made for robust or a 'successful and consistent' transition protocol [4-6]. There is currently a lack of joint planning and commissioning and the need to improve this, specifically between children and adult services, was recognised. In order to facilitate this the key responsibilities of commissioners were described as (i) developing service specifications based on local needs assessments; (ii) stimulating the market place to fill identified gaps in service provision and (iii) stimulating providers to provide best practice services based on research evidence [5]. CQUIN (Commissioning for Quality and Innovation) was suggested as a mechanism to aid effective commissioning practice [4]. Further, the need for a transition protocol to embed the principles of personalised planning as part of the personalisation agenda was highlighted [6]. Finally, there was a practical suggestion that the management of transition in hospitals, primary care and community care should be factored into the overall rating which is calculated as part of CQC inspection models, in order to incentivise improved performance.

1.2 Clinic structure

The 'age conundrum' was identified as a barrier to successful transition [4]. This means that at age 18 a young person should be treated in adult services but for children and young people with complex health needs, including learning difficulties and mental health difficulties, this is not necessarily the most developmentally appropriate age to transfer to adult health care providers. Further, age boundaries were perceived to be set arbitrarily and as such considerable variety and inconsistencies existed between different services. There was variation in suggestions for how this 'age boundary' challenge should be addressed. Some suggested a distinct young person's service, e.g. spanning age 14-25; whilst others suggested a flexible approach to managing transition within the existing structures [7]. Development of young person focussed services in the community were also suggested to provide additional support for children from 11-25 [8].

1.3 Funding

Funding for transition was noted to be complex, fragmented and often not clear. In order to improve this it was suggested that funding responsibilities for equipment and short-term breaks should be agreed earlier between commissioners and providers in order to avoid delays during transition [7]. Current funding for adolescent health and social care was deemed

insufficient, particularly with respect to developmental disorders and mental health, where workloads were perceived to be rising amidst inadequate staffing and resources [4].

Upstream investment, in terms of the commissioning of evidence based preventative measures, and early stage intervention in children and young people services were advocated. This shift in investment was cited as having the potential to enable the child or young person to receive appropriate services more swiftly and for a shorter time and as a means to achieve future savings [4].

The implication of a lack funding was that there may be limited available services suitable for young adults including: a lack of age appropriate short break facilities; few chances of meaningful employment; few residential places that can take young people with very complex health needs; and few health specialists with a holistic approach to a young person's care [5].

1.4 Co-ordination

Appleton [4,p19] argues that effective commissioning practice depends on important relationships between key groups. "Engagement between commissioners and local clinicians, as well as with young people and their families will be increasingly important in the new commissioning environment." Building relationships has been highlighted in a number of reports as a critical factor in successful transition planning. The role of commissioners in helping services to join up could include facilitating a local Transitions Forum, including representatives from children's and adult's services, the voluntary sector and service user groups to review and monitor transition protocols, and providing an arena for review and service development.

"Current commissioning models often place CAMHS commissioning and AMHS within different frameworks, structures and organisations. Not facilitated joint working across the two sectors and has not enabled a sharing of ideas and solutions. As a result, separate service development has taken place that has not properly addressed the issues relating to transition" [4,p10] Commissioners should ensure that joint agency planning and commissioning enable the development of person centred, co-ordinated and integrated packages of care and support for children and young people, and the smooth transition to adult services and avoid crises [9].

2 Organisation and delivery of care

Although this literature review was focussed on commissioning for transition, a number of the papers we reviewed contained information or recommendations related to the organisation and delivery of care. An understanding of what good organisation and delivery of care may be helpful for commissioners to inform commissioning strategies. As such, a brief summary of the key topics which emerged are noted here. The four sub-themes related to organisation and delivery were: transition plan, health professionals, holistic care, and coordination.

2.1 Transition plan (TP)

Transition planning, which should be stated and updated in a document shared with the young person, should: start early and continue if necessary into adult care. It should be tailored to the needs of young person and family and be age and developmentally appropriate. The TP should also include education, social services and voluntary agencies as active partners [5,p40 7 9]. Transition planning is not happening everywhere and opportunities are being missed to focus on employment and housing options at an early stage, for example by embedding career planning in person-centred transition pathways [10]. There is debate about whether a health passport is implied in a TP or whether it should be a separate document [7 11].

2.2 Health professionals

The literature reviewed here referred to three different health professionals involved in transition: the GP, key worker, and coordinator. It was noted that “the adult sector and adult physicians may have little experience in ‘paediatric’ diseases in adult life” and as such the involvement of GPs at an earlier stage was recommended for future service planning. [7]

A key worker was described as a person who assists an individual to negotiate transition which all transition patients should have [7 9 11]. Co-ordination of transition care is critical and, if this does not already exist, a key worker should be identified for each young person to oversee their transition, ensuring links with a counterpart within the receiving adult service [5].

A coordinator is a person working in a service that involves Transition, who works at a managerial level to ensure effective processes which might include: reminding young people by text of forthcoming clinics, ensuring transition plans are drawn up, the appointment of a named key worker. Best practice for managing transition should be followed [7 9].

2.5 Holistic care

To support holistic care transition should include arrangements for: Independent living, employment, health and social inclusion [9,p41 11]. Key characteristics of an effective holistic transition process include: young person centred, wide age range, informal, flexible, voluntary, confidential, free, independent, early intervention, continuity, etc. During transition there should be support of multiple needs: emotional; mental; personal; physical; social welfare; practical; and safeguarding with complementary interventions delivered under one roof [12].

2.4 Effective co-ordination

To support the delivery of a regional multi-agency transition strategy, there should be a transition lead in every region, supported by an implementation group involving all key stakeholders, in particular young people and their families[10]. In order to be truly effective CCGs and Local Authorities should actively listen and learn from young people and their families' experiences [7]. One of the five ambitions, promoted by Youth Access, is that "services will be integrated and care will be coordinated around the individual, with an optimal experience of transition to adult services for those young people who require ongoing health and care in adult life." [8,p3]

3 Conclusion

The purpose of this grey literature review was to supplement a systematic review of academic literature, as described in Figure S1, which failed to identify any papers meeting the criteria which required a focus on commissioning for healthcare transition. This has been identified as a gap in the academic literature. The grey literature reviewed did not provide sufficient detail, nor consensus to allow rigorous review or theming of content, however it has provided some useful information and opinions on key topics such as transition protocols, clinic structures, funding and coordination. We hope this review is of use and interest to academics and other professionals working in this area.

References

1. New Frontiers in Grey Literature. Fourth International Conference on Grey Literature; 1999; Washington D.C. USA. GreyNet.
2. Wealth Sciences Library. Grey Literature. Wealth Sciences Library: University of Washington, 2014.
3. Levy Y, Ellis T. A Systems Approach to Conduct an Effective Literature Review in Support of Information Systems Research. Informing Science 2006;**9**:181-212.
4. Appleton S, Pugh K. Planning Mental Health Services for Young Adults –Improving Transition: A Resource for Health and Social Care Commissioners. In: Unit. NMHD, ed., 2011.
5. Department of Health. Better Care: Better Lives: Improving outcomes and experiences for children, young people and their familiaries living with life-limiting and life-threatening conditions. London: Department of Health, 2008.
6. Department of Health. Equity and excellence: Liberating the NHS. London: Department of Health, 2010.
7. CQC. From the pond into the sea: children's transition to adult health services. . In: Commission CQ, ed. London, 2014.
8. Youth Access. Making integration a reality. Part 1 - joining up the commissioning across young people's services across health, social care, housing and youth services. Commissioning Briefing: Young People's Health Partnership, 2014.
9. RCGP., RC PSYCH. Improving the Health and Wellbeing of People with Learning Disabilities: An Evidence-Based Commissioning Guide for Clinical Commissioning Groups (CCGs). In: Royal College of General Practitioners RCoP, Learning Disabilities Observatory, ed. London: http://www.improvinghealthandlives.org.uk/securefiles/170405_1846//RCGP%20LD%20Commissioning%20Guide%202012%2010%2009%20FINAL.pdf, 2012.
10. Department of Health. Valuing people now: a new three-year strategy for people with learning disabilities. Making it happen for everyone. London: Department of Health, 2009.
11. National Autistic Society. Discussion paper: Improving commissioning standards in services for ASD Patients. London: National Autistic Society, 2006.
12. Youth Access. Making integration a reality. Part 2 - developing effective holistic services for young people in transition. . London: Young People's Health Partnership, 2014.
13. Together for Short Lives. Commissioning children's palliative care: a guide for Clinical Commissioning Groups (CCGs). London: Together for Short Lives, 2013.
